# Supplementary material for: Benefit of an Ultrasonic Irradiation on the Depollution by Washing of Nickel- or Zinc-Contaminated Vermiculite
Source: Molecules. 2025 Feb 28;30(5):1110. doi: 10.3390/molecules30051110 (PMC11901782; doi:10.3390/molecules30051110)
Supplement: Supplementary file 1 [file molecules-30-01110-s001.zip › molecules-3481079-supplementary.pdf]

Article

Benefit of an Ultrasonic Irradiation on the Depollution by Washing of Nickel- or Zinc-Contaminated Vermiculite

Antoine LEYBROS[1], Sophie HERR[1,2], Rita SALAMEH[1,2] and Rachel PFLIEGER[2]

[1]CEA, DES, ISEC, DMRC, Univ Montpellier, Marcoule 30207, France

[2]ICSM, Univ Montpellier, CEA, CNRS, ENSCM, Marcoule 30207, France

Supplementary Information

Table S1. Preparation of zinc (a) and nickel (b) contaminated vermiculite batchs: operating conditions for adsorption conditions and adsorbed zinc and nickel concentration in soil.

a)

Batch	Amount of sorbed metal ($mg.g^{-1}$)	Vermiculite mass (g)	Treatment duration (weeks)	Zn solution concentration ($mg.L^{-1}$)	Zn solution volume (L)
B3.1(Zn)	41.8	20	2	450	2
B3.2(Zn)	39.7	20	2	450	2
B3.3(Zn)	38	20	2	450	2
B3.4G(Zn)	37.5	100	2	3200	1
B3.5G(Zn)	37.5	100	2	3200	1
B4.0(Zn)	35.5	10	1	450	1

b)

Batch	Amount of sorbed metal ($mg.g^{-1}$)	Vermiculite mass (g)	Treatment duration (weeks)	Zn solution concentration ($mg.L^{-1}$)	Zn solution volume (L)
B3.1(Ni)	37.4	20	2	450	2
B3.2(Ni)	33.5	20	2	450	2
B3.3(Ni)	33	20	2	450	2

B3.4G(Ni)	30.2	100	2	2300	1
B3.5G(Ni)	31.9	100	2	2300	1

Table S2. Desorption of zinc (a) and nickel (b) from vermiculite by washing in silent conditions or by combination of washing and ultrasound irradiation: operating conditions and results.

a)

Run	Batch	Amount of sorbed metal ($mg.g^{-1}$)	Treatment conditions	Vermiculite mass/leachant volume ($g.L^{-1}$)	Duration (h)	Desorption yield (%)
Zn-1	B3.2(Zn)	39.7	20 kHz – HCl (0.1 M)	20	3	79
Zn-2	B3.5(Zn)	37.5	362 kHz – HCl (0.1 M)	20	1	78
Zn-3	B4.0(Zn)	35.5	H-silent – HCl (0.1 M)	20	3	74
Zn-4	B3.4G(Zn)	37.5	V-silent – HCl (0.1 M)	20	1	73
Zn-5	B3.2(ZN)	39.7	H-silent – HCl (0.1 M)	10	3	80
Zn-6	B3.2(Zn)	39.7	H-silent – HCl (0.1 M)	50	3	68
Zn-7	B3.1(Zn)	41.8	H-silent – HCl (0.1 M)	100	3	53
Zn-8	B3.5G(Zn)	37.5	362 kHz – HCl (0.1 M)	50	1	76
Zn-9	B3.5G(Zn)	37.5	362 kHz – HCl (0.1 M)	100	1	56
Zn-10	B3.2(Zn)	39.7	20 kHz – HCl (0.1 M)	10	3	83
Zn-11	B3.2(Zn)	39.7	20 kHz – HCl (0.1 M)	50	3	69
Zn-12	B3.5G(Zn)	37.5	V-silent – HCl (0.1 M)	50	1	75
Zn-13	B3.5G(Zn)	37.5	362 kHz – HCl (0.1 M)	20	0.08	66
Zn-14	B3.5G(Zn)	37.5	V-silent – HCl (0.1 M)	20	0.08	67
Zn-15	B3.5G(Zn)	37.5	362 kHz – HCl (0.1 M) + $MgCl_2$ (1 M)	20	1	86
Zn-16	B3.5G(Zn)	37.5	V-silent – HCl (0.1 M) + $MgCl_2$ (1 M)	20	1	85

Zn-17	B3.1(Zn)	41.8	20 kHz – citric acid (0.5 M)	10	6	87
Zn-18	B3.5G(Zn)	37.5	362 kHz – citric acid (0.5 M)	10	3	88
Zn-19	B3.1(Zn)	41.8	H-silent – citric acid (0.5 M)	10	6	81
Zn-20	B3.1(Zn)	41.8	362 kHz – HCl (0.1 M)	10	3	85

b)

**Run**	**Batch**	**Amount of sorbed metal ($mg.g^{-1}$)**	**Treatment conditions**	**Vermiculite mass/leachant volume ($g.L^{-1}$)**	**Duration (h)**	**Desorption yield (%)**
Ni-1	B3.2(Ni)	33.5	20 kHz – HCl (0.1M)	20	3	21
Ni-2	B3.4G(Ni)	30.2	362 kHz – HCl (0.1M)	20	3	29
Ni-3	B3.4G(Ni)	30.2	H-silent – HCl (0.1 M)	20	3	17
Ni-4	B3.4G(Ni)	30.2	V-silent – HCl (0.1 M)	20	3	24
Ni-5	B3.2(Ni)	33.5	H-silent – HCl (0.1 M)	10	3	25
Ni-6	B3.4G(Ni)	30.2	H-silent – HCl (0.1 M)	50	3	15
Ni-7	B3.4G(Ni)	30.2	362 kHz – HCl (0.1 M)	10	3	29
Ni-8	B3.4G(Ni)	30.2	362 kHz – HCl (0.1 M)	50	3	16
Ni-9	B3.4G(Ni)	30.2	362 kHz – HCl (0.1 M)	100	3	11
Ni-10	B3.2(Ni)	33.5	20 kHz – HCl (0.1M)	10	3	28
Ni-11	B3.2(Ni)	33.5	20 kHz – HCl (0.1 M)	50	3	20
Ni-12	B3.5G(Ni)	31.9	V-silent – HCl (0.1 M) + $MgCl_2$ (1 M)	20	3	20
Ni-13	B3.5G(Ni)	31.9	362 kHz – HCl (0.1 M) + $MgCl_2$ (1 M)	20	3	27
Ni-14	B3.5G(Ni)	31.9	20 kHz – HCl (0.1 M) + $MgCl_2$ (1 M)	20	3	38
Ni-15	B3.1(Ni)	37.4	20 kHz – citric acid (0.5 M)	10	6	13

Ni-16	B3.5G(Ni)	31.9	362 kHz – citric acid (0.5 M)	10	3	13
Ni-17	B3.1(Ni)	37.4	H-silent – citric acid (0.5 M)	10	6	11
Ni-18	B3.5G(Ni)	31.9	V-silent – citric acid (0.5 M)	10	3	10

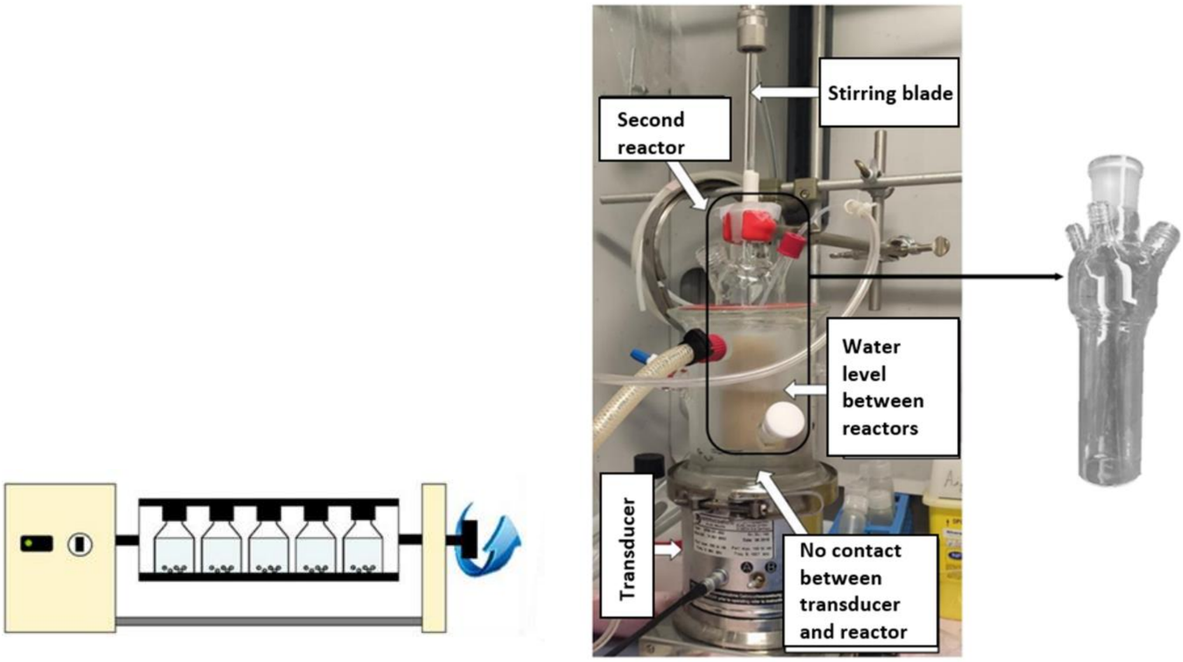

a) b)

Figure S1 Schematic diagram of experimental setup used in silent conditions. (a) H-silent conditions and (b) V-silent conditions.

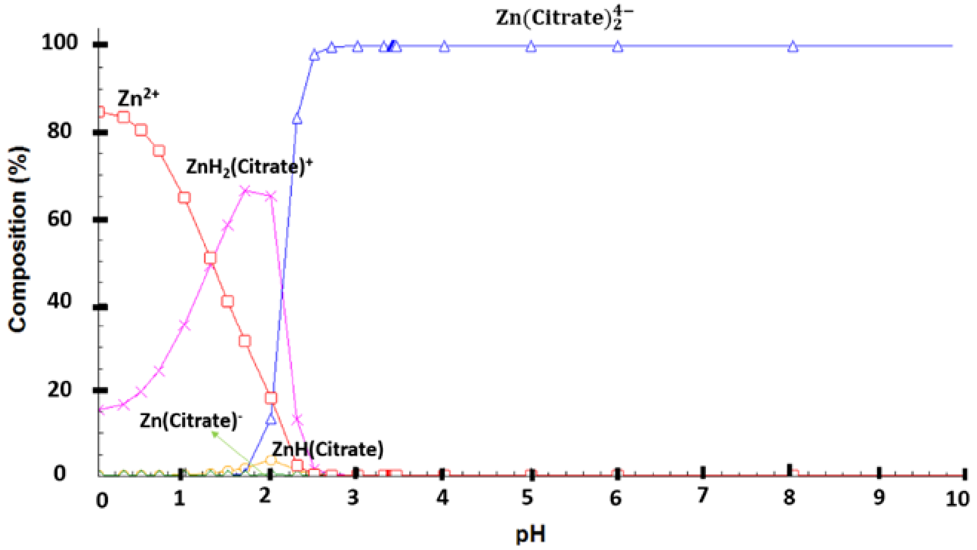

a)

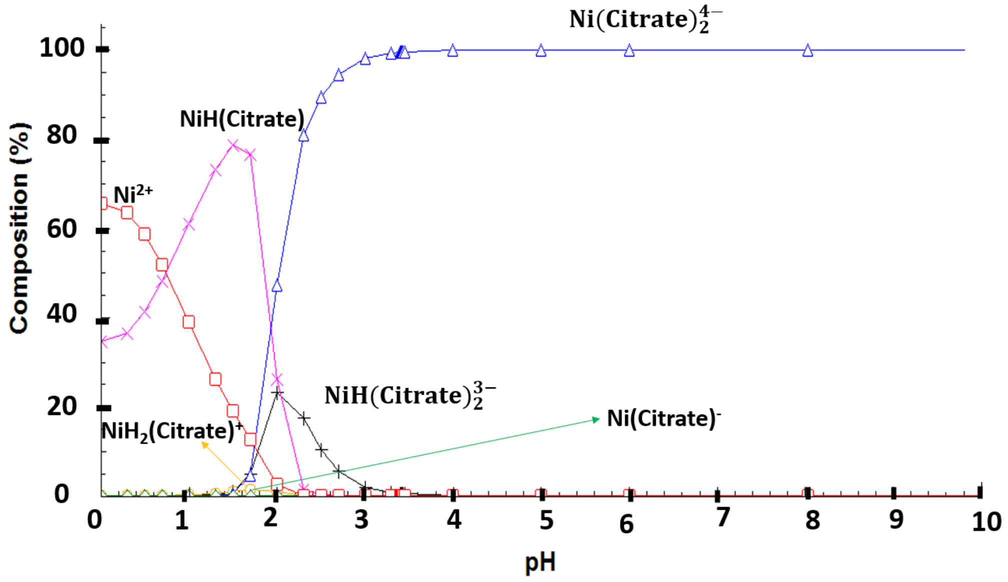

b)

Figure S2. Speciation diagrams of Zn^{2+} and Ni^{2+} calculated using PhreeqC for a solution containing (a) 380 mg/L Zn^{2+} or (b) 350 mg/L Ni^{2+} and 0.5 M citric acid.
